# Tetrahedral Clusters Stabilized by Alloying

**DOI:** 10.1021/acs.jpca.3c06033

**Published:** 2023-12-19

**Authors:** Cesare Roncaglia, Riccardo Ferrando

**Affiliations:** † Dipartimento di Fisica dell’Università di Genova, via Dodecaneso 33, Genova 16146, Italy; ‡ CNR-IMEM, via Dodecaneso 33, Genova 16146, Italy

## Abstract

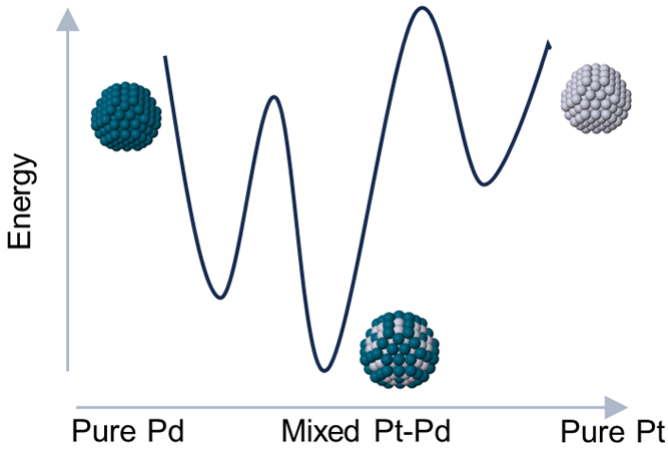

A family of nanoclusters of tetrahedral symmetry is proposed. These clusters consist of symmetrically truncated tetrahedra with additional hexagonal islands on the four facets of the starting tetrahedron. The islands are placed in stacking fault positions. The geometric magic numbers of these clusters are derived. Global optimization searches within an atomistic potential model of Pt–Pd show that the tetrahedral structures can be stabilized for intermediate compositions of these nanoalloys, even when they are not the most stable structures of the elemental clusters. These results are also confirmed by density functional theory calculations for the magic sizes 59, 100, and 180. A thermodynamic analysis by the harmonic superposition approximation shows that Pt–Pd tetrahedral nanoalloys can be stable even above room temperature.

## Introduction

Many of the properties of metallic nanoparticles (NPs) depend on the arrangement of their atoms.^1^ The most common shapes for face-centered cubic metal nanoparticles are truncated octahedra,^2^ decahedra (Dh), and icosahedra.^3,4^ Recently, other types of structures based on different symmetries have been the object of intense research due to their unique properties. A remarkable example is given by nanoparticles exhibiting tetrahedral (Th) symmetry.^5−8^ These NPs have shown good catalytic^9−14^ as well as optical^15−17^ properties. Moreover, tetrahedra are the building blocks of multitwinned NPs, such as decahedra and icosahedra;^18^ their study, then, is of primary importance. Unfortunately, metal nanoparticles showing tetrahedral symmetry are known to be energetically favored only for very small sizes and unstable for larger sizes, a feature that constitutes a drawback for practical applications.

As a matter of fact, tetrahedral structures are known to be stable (i.e., to be the lowest-energy structures) at a density functional theory (DFT) level of accuracy, only up to a few atoms, i.e., the famous case of Au_20_.^19^ Larger tetrahedra can grow as nonequilibrium, metastable structures starting from octahedral seeds.^20^ Another notable tetrahedral structure was found by Leary and Doye in 1999.^21^ In that work, they found that a tetrahedral structure with *N* = 98 atoms is the global minimum for the Lennard-Jones atomistic potential. The Leary tetrahedron can be constructed by building a 19-atom tetrahedron (which is a 20-atom regular tetrahedron without 1 vertex) on each of the 4 facets of a regular 20-atom tetrahedron for a total of 56 atoms. The remaining 42 atoms are placed in 6 hexagonal patches on the naked edges of the starting 20-atom tetrahedron. The stability of the Leary tetrahedron was also proven for some compositions of the Pt–Pd Gupta atomistic force field,^22^ but not confirmed at the DFT level, for which an attempt was made by considering Au–Pd clusters.^23^

In this work, we show that a different family of structures based on tetrahedral symmetry can be stabilized even at the DFT level up to *N* = 180 atoms (∼1.6 nm), thanks to the mixing of Pd and Pt metals. Therefore, our calculations indicate that some tetrahedral metal NPs actually represent the lowest-energy structures in a size range that is far wider than what is usually thought. Other than that, we remark that Pt–Pd nanoparticles owe their interest to the exceptional catalytic activity that was shown to be superior than that of pure metals for several reactions.^24−27^

## Theoretical Methods

In this section, we describe the theoretical methods used. Global optimization searches were done by using our own basin-hopping code,^28,29^ in which atomic interactions are approximated by the Gupta atomistic potential, which we describe in the following section. Density functional theory was used to estimate the energy of the structures found in the global optimization searches. Finally, the harmonic superposition approximation was used to prove the stability of tetrahedral nanoparticles at temperatures different from 0 K.

### Atomistic Potential

The Gupta potential energy of a nanoparticle can be written as a sum of single atomic contributions
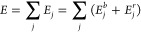
1where
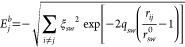
2is the negative binding term due to attractions and
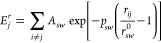
3models the positive repulsive term. Here, *r*_*ij*_ is the distance between atoms *i* and *j*, and *s*(*w*) refers to the chemical species of atom *i*(*j*). If *s* = *w*, then *r*_*sw*_^0^ is the nearest-neighbor distance in the corresponding bulk lattice, while for *s* ≠ *w*, *r*_*sw*_^0^ is taken as the arithmetic mean of the distances of pure metals. Interactions between pairs of atoms are cut off in order to truncate the sums. In particular, exponentials in eqs 2 and 3 are replaced by fifth-order polynomials, of the form , between distances *r*_*c*1_ and *r*_*c*2_ (which are second- and third-neighbor distances in the bulk lattice, respectively), with *a*_3_, *a*_4_, and *a*_5_ fitted in each case to obtain a function which is always continuous, with first and second derivatives for all distances, and goes to zero at *r*_*c*2_. Parameters for Pt–Pd can be found in ref (30).

### Density Functional Theory

Density functional theory was used to estimate the total energy of a cluster by making a relaxation of its coordinates, as given by the global optimization searches. All calculations were made by using the Quantum Espresso open-source software.^31^ We used two different exchange-correlations functionals: Perdew–Burke–Ernzerhof (PBE)^32^ and the local density approximation (LDA).^33^ The convergence thresholds for the total energy, for the total force, and for electronic calculations were set to 10^–4^ Ry, 10^–3^ Ry/a.u., and 5 × 10^–6^ Ry, respectively. We used a periodic cubic cell whose size was set to 25 Å for *N* = 59 NPs and 30 Å for *N* = 100 and 180 NPs. Cutoffs for the wave function and charge density were set as suggested by the following pseudopotentials that we used: Pt.pbe-n-kjpaw_psl.1.0.0.UPF, Pd.pbe-n-kjpaw_psl.1.0.0.UPF, Pt.pz-n-kjpaw_psl.1.0.0.UPF, and Pd.pz-n-kjpaw_psl.0.2.2.UPF for PBE and LDA, respectively. They are currently available at https://pseudopotentials.quantum-espresso.org/legacy_tables/ps-library/ and https://dalcorso.github.io/pslibrary/.

### Harmonic Superposition Approximation

The harmonic superposition approximation^34^ was used to approximate the partition function of a nanoalloy in order to estimate the free energy as a function of temperature. Let *s* denote a local minimum of the energy landscape of a PtPd nanoalloy, which corresponds to a locally stable structure such as Dh or Th. In the HSA, its free energy *F*_*s*_ is given by the sum of translational, symmetry, vibrational and rotational contributions added to the energy *E*_*s*_ of the local minimum *s*:
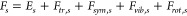
4The term *F*_*tr*,*s*_ due to translation is independent of the structure so that in free-energy differences it may be neglected. The other terms are given by
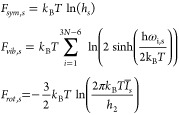
5where *h*_*s*_ is the order of the symmetry group, ω_*i*,*s*_ represents the nonzero normal-mode frequencies, and  is the geometric average of the principal moments of inertia . Normal-mode frequencies were calculated by using the atomistic Gupta potential only since within DFT such a calculation is quite expensive, especially for larger sizes.

## Results and Discussion

**Figure 1:**
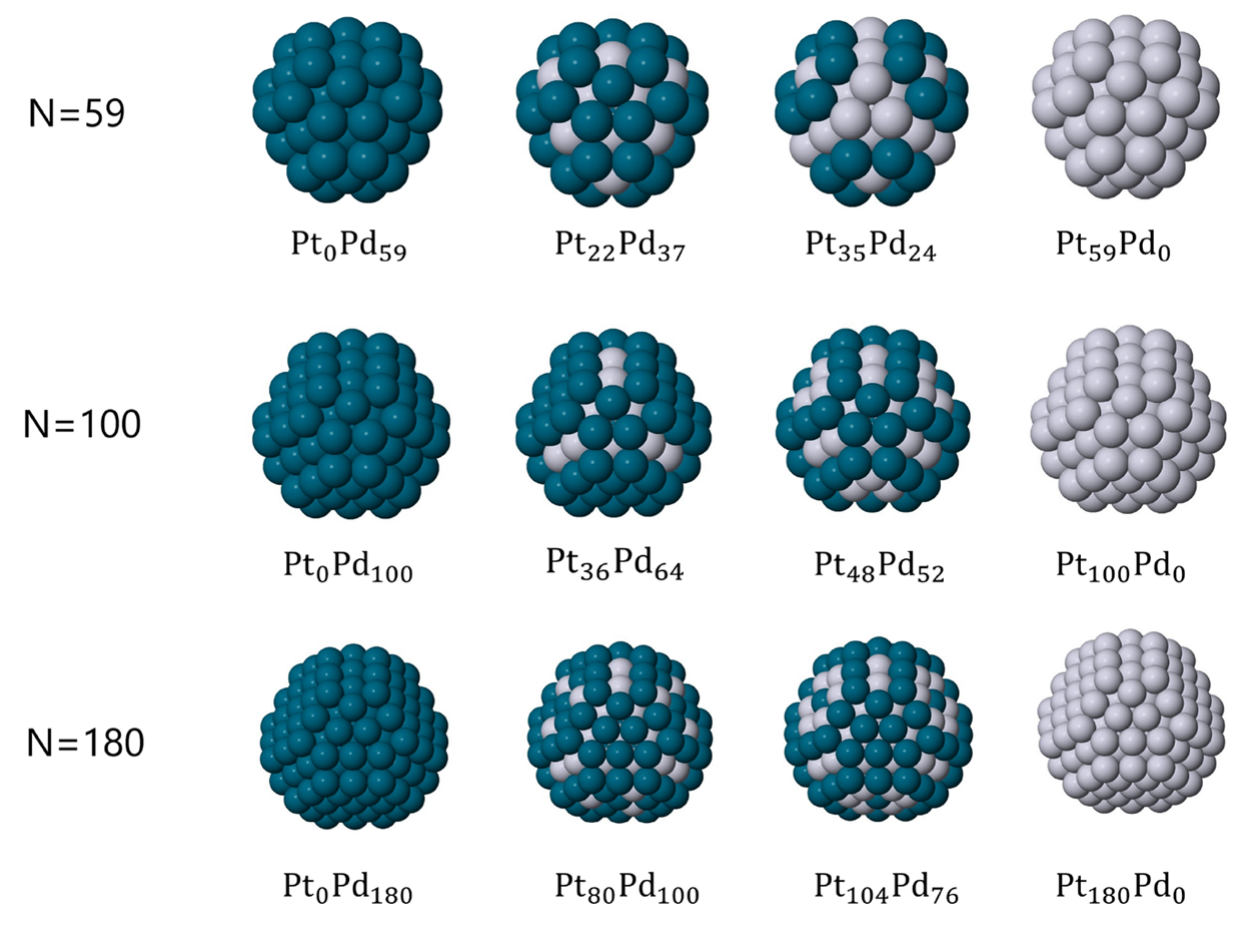
Examples of truncated tetrahedra with both regular and irregular stacking fault islands.

**Figure 2:**
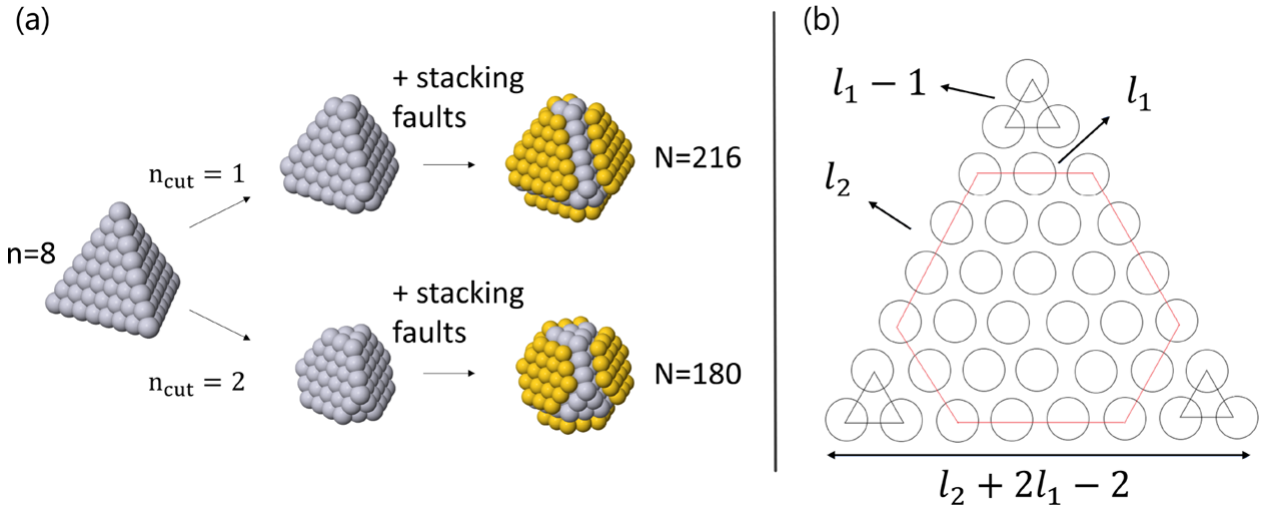
(a) Two examples of different cuts from the same tetrahedral seed and (b) schematic representation of an irregular hexagon for the calculation of the number of atoms in stacking fault islands.

The family of structures that is the object of this research is composed of truncated tetrahedra that have four stacking fault islands on their facets. Some examples are shown in Figure 1 for different sizes (*N* = 59, 100, and 180) and compositions. The structure with *N* = 59 atoms (see Figure 1) can be constructed by truncating the four vertices from the regular 34-atom tetrahedron and by adding four regular hexagonal patches as stacking faults on each of the four facets. This structure was already found by Doye and Wales in 1995^35^ as the global minimum for the Morse atomistic potential. The structure with *N* = 100 atoms (see Figure 1) was previously found by Manninen and Manninen in 2002 as the global minimum for two atomistic models based on the coordination numbers.^36^ This structure can be obtained by truncating the four vertices from the 56-atom regular tetrahedron and completed by adding four irregular hexagons as stacking fault islands on each of the four facets. We did not find any result for tetrahedral structures for *N* = 180 in the literature. This structure, see Figures 1 and 2a, is built by cutting four 4-atom tetrahedra from the apexes of the regular 116-atom tetrahedron and by finally placing four regular hexagonal patches as the stacking fault on the four facets. We note that for all mixed compositions, the arrangement of the two chemical species is always the same. In particular, Pt atoms lie almost entirely in the core of the nanoparticles, whereas Pd atoms tend to occupy the surface shell where they can accommodate low-coordination sites; ultimately, they also occupy some of the inner sites in the center of the NPs. Eventually, as can be seen in Figure 1, Pt atoms in excess are located inside the hexagonal stacking fault islands. The surface segregation of palladium is consistent with previous numerical simulations^37−40^ and experiments.^41−44^ To our knowledge, the stability of any of these structures at the DFT level was never proven. In the following section, we derive the new series of magic numbers for these structures, referring to Figure 2 for a schematic visualization of the proof.

In fcc stacking, a regular tetrahedron with a given edge of *n* atoms is composed of *n* equilateral triangles with edges of increasing size from 1 (the vertex) to *n* (the base). Thus, the total number of atoms in a tetrahedron is given by
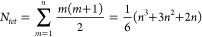
6since the number of atoms in an equilateral triangle having *m* atoms in its edge is exactly *m*(*m* + 1)/2. If at each vertex of the tetrahedron a cut of length *n*_*cut*_ is made, then the total number of atoms in a regularly truncated tetrahedron is therefore given by
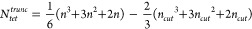
7

Stacking faults can be either regular or irregular hexagons. For the calculation of the number of atoms of an irregular hexagon, we refer to Figure 2b. Let *l*_1_ and *l*_2_ be the two side lengths of the irregular hexagon. Then, to calculate the total number of atoms, it is sufficient to take the size of the triangle and subtract 3 times the size of the small triangles created by the cuts. Then
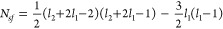
8is the number of atoms in an irregular-hexagon stacking fault island. If we place such an island on one of the four facets of the truncated tetrahedron, as in Figure 2a, then we have to make the substitutions *l*_1_ – 1 = *n*_*cut*_ and *l*_2_ = *n* – 2*n*_*cut*_ – 1. This is equivalent to say
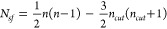
9

Finally, we are given the number of regularly truncated tetrahedrons with irregular stacking fault islands
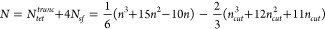
10which gives, for example, *N* = 100 for *n* = 6 and *n*_*cut*_ = 1 and *N* = 116 for *n* = 7 and *n*_*cut*_ = 2.

Regular hexagons in stacking fault islands are obtained when *l*_1_ = *l*_2_ or *n*_*cut*_ = (*n* – 2)/3. In this case, the total number of atoms is given by
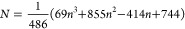
11which gives the magic series *N* = 59, 180, 394, ... for *n* = 5, 8, 11, ...

**Figure 3:**
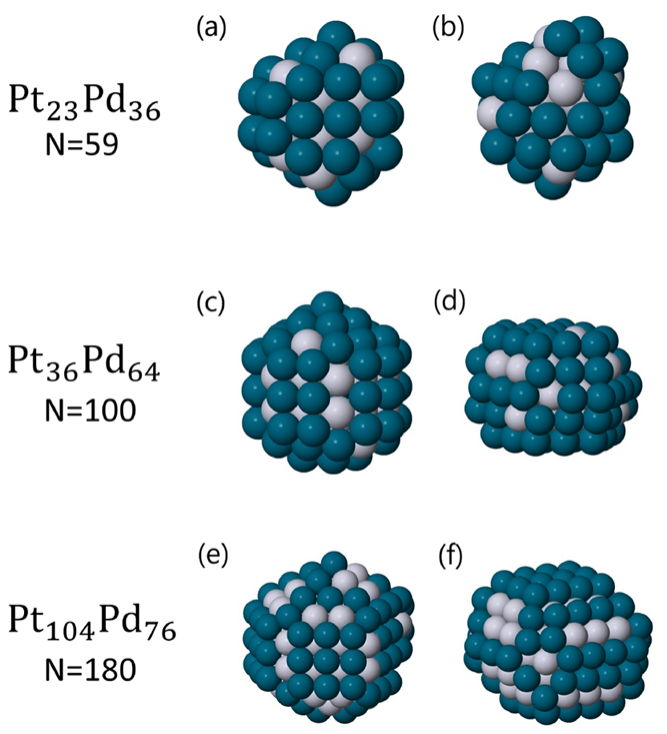
(a) Decahedral and (b) icosahedral NP for Pt_23_Pd_36_. (c) Decahedral and (d) twin structure for Pt_36_Pd_74_. (e) Decahedral and (f) twin structure for Pt_104_Pd_76_.

**Figure 4:**
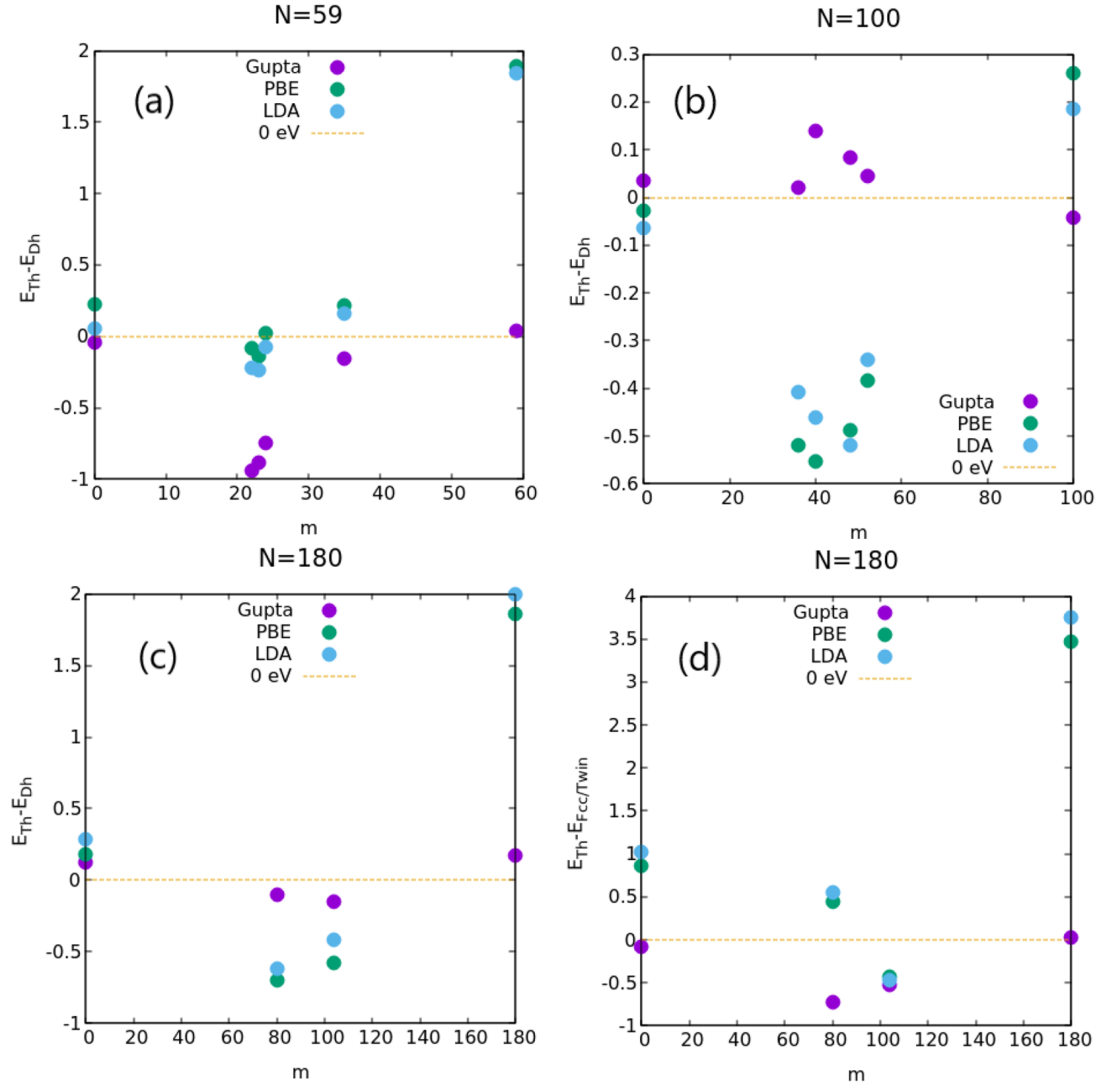
Energy differences between the best tetrahedron and best decahedron for (a) *N* = 59, (b) *N* = 100, and (c) *N* = 180. (d) Energy differences between the best tetrahedron and best fcc/twin structure for *N* = 180. For all sizes and compositions, we considered the atomistic Gupta potential and two different exchange-correlation functionals: PBE and LDA.

In order to assess the stability of these magic tetrahedral clusters, we first performed unseeded and seeded global optimization searches using a Gupta atomistic potential. We considered Pt_*m*_Pd_*N*–*m*_ nanoalloys with *N* = 59, 100, and 180. In particular, for *N* = 59 we set *m* = 0, 22, 23, 24, 35, 59, for *N* = 100 we set *m* = 0, 36, 40, 48, 52, 100, and for *N* = 180 we set *m* = 0, 80, 104, 180. During global optimizations, the exploration of the potential energy surface of the systems also allowed us to collect other structures that are in competition, i.e., close in energy, with truncated tetrahedra. The main competing structural motif is decahedral. Some examples of this and other competing structures can be found in Figure 3. Subsequently, we performed DFT relaxations of the competing cluster coordinates found by the global optimization searches. Finally, we computed energy differences for all of the sizes and compositions studied. The results of all calculations are summarized in Figure 4; numerical values are reported in Table S15 in the Supporting Information. For pure metals, decahedra and tetrahedra are always in competition for the Gupta potential (|Δ*E*| < 0.05 eV) but not for DFT calculations, for which Dh are always favored consistently for both exchange-correlation functionals. The only exception is Pt_0_Pd_100_, for which even at the DFT level the two structural motifs are in close competition. For mixed compositions, tetrahedra are generally stabilized. In the case of *N* = 59, tetrahedra are favored for two of the four compositions tested: Pt_22_Pd_37_ and Pt_23_Pd_36_. For the Pt_24_Pd_35_ composition, the Gupta potential strongly favors the tetrahedral motif, while DFT calculations agree with respect to their competition (|Δ*E*| < 0.08 eV for both exchange-correlation functionals). Finally, only in the case of Pt_35_Pd_24_, decahedra are consistently favored at the DFT level, in contrast with the atomistic calculation. In the case of *N* = 100, tetrahedra are strongly favored at the DFT level, whereas the Gupta potential tends to prefer the decahedral motif but still with a small energy difference. Finally, for larger NPs (*N* = 180), tetrahedra are consistently favored at both atomistic and DFT levels for at least one composition, i.e., Pt_104_Pd_76_. For the other mixed composition, the face-centered cubic motif is preferred at the DFT level, with the tetrahedron being favored instead by the atomistic potential.

Mixing energy differences were also calculated to analyze some of the data reported in Table S15. The results and plots are reported in Figures S1–S3 in the Supporting Information.

**Figure 5:**
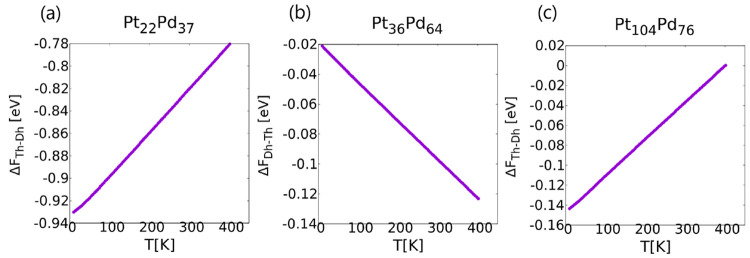
Free-energy differences for one mixed composition at each size. (a) Pt_22_Pd_37_ for *N* = 59, (b) Pt_36_Pd_64_ for *N* = 100, and (c) Pt_104_Pd_76_ for *N* = 180.

The stability of some of the previously shown Dh and Th structures was studied for temperatures other than 0 K by estimating free energy differences thanks to the HSA. Results for the free-energy differences between Dh and Th are shown in Figure 5. We measure free-energy differences from the structure having a lower potential energy: Δ*F*_*a*–*b*_ = *F*_*a*_ – *F*_*b*_ where *E*_*a*_ < *E*_*b*_.

For all cases, the entropic effects tend to stabilize the decahedral motif with increasing temperatures. In fact, Δ*F* = *F*_*Th*_ – *F*_*Dh*_ increases when Th is favored (Figure 5(a) and (c)) at 0 K, and Δ*F* = *F*_*Dh*_ – *F*_*Th*_ decreases when Dh is favored at 0 K (Figure 5(b)). We notice, however, that for the case of Pt_22_Pd_37_ and Pt_104_Pd_76_, the increase in Δ*F* is not enough to change its sign, so Δ*F* < 0 for all temperatures at least up to room temperature. This means that, at least for the atomistic model, we can conclude that the truncated tetrahedron remains the most stable structural motif, even in this temperature range. We performed a short molecular dynamics run of 1 *μs* at a constant temperature of 400 K for the three tetrahedral structures considered for free-energy differences. In all three simulations, we did not observe any transition. Energy plots and simulation details are reported in the Supporting Information. We speculate that the order of magnitude of entropic contributions in free-energy differences (∼0.1 eV at *T* = 300 K) could also be the same for DFT calculations. In fact, we performed DFT calculations for normal-mode frequencies for a small Pt_2_Pd_4_ cluster, and we found good agreement between numerical values. Results and details for the calculations are reported in the Supporting Information.

A question that arises naturally concerns the causes of the stabilization of tetrahedral nanoparticles induced by the alloying of the two metals. It is our belief that the main reason for this result originates from a combined effect of (i) the limited availability of competing shapes other than tetrahedra at a given size and (ii) the choice of the right composition for the tetrahedral shape. In fact, by properly selecting the right amount of the two metals, one can build the tetrahedral nanoparticles in such a way that all palladium atoms decorate the four stacking fault islands—entirely or partially, as can be seen in Figure 1—as well as the four triangular facets of the tetrahedron that are left exposed after the cut of the vertices. Some of the palladiums may also be included in the central sites of the NPs. This chemical arrangement is the best for these tetrahedral shapes. In addition, it turns out that such a composition is not the best one for the other competing shapes, such as decahedra and twin structures. For example, even for the decahedral shape at size *N* = 100, which is a very good one since it is missing only one vertex from the perfectly symmetric 101-atom Marks decahedron, the four compositions used for our calculations, Pt_36_Pd_64_, Pt_40_Pd_60_, Pt_48_Pd_52_, and Pt_52_Pd_48_ are not the optimal ones for decorating the decahedral shape.

In order to gain more quantitative insights into the possible reasons for the stabilization of tetrahedral nanoparticles, we calculated the occurrence of Pt–Pt, Pt–Pd, and Pd–Pd bonds as well as coordination numbers for atoms in some of the competing isomers. The results of the calculations for the number of different bonds are reported in Table 1.

**Table 1:** Number of Pt–Pt, Pd–Pd, and Pt–Pd Bonds for Th and Dh Structures in Pt_22_Pd_37_, Pt_52_Pd_48_, and Pt_104_Pd_76_

Bond type	Dh	Th
	Pt_22_Pd_37_	
Pt–Pt	66	60
Pd–Pd	69	60
Pt–Pd	105	120
Pt_52_Pd_48_		
Pt–Pt	192	198
Pd–Pd	70	72
Pt–Pd	177	168
Pt_104_Pd_76_		
Pt–Pt	418	432
Pd–Pd	115	120
Pt–Pd	319	288

In Pt_22_Pd_37_, both decahedral and tetrahedral structures have a total of 240 bonds. The truncated tetrahedron has 60 Pt–Pt and 60 Pd–Pd bonds and 120 Pt–Pd bonds. The decahedral isomer has 66 Pt–Pt bonds, 69 Pd–Pd bonds, and 105 Pt–Pd bonds. Two atoms are bonded if their distance is within 20% of the nearest-neighbors distance. For Pt–Pd bonds, the nearest-neighbor distance is the arithmetic average of Pt–Pt and Pd–Pd nearest-neighbor distances. We used *d* = 1.385 and 1.375 Å for Pt–Pt and Pd–Pd respectively. We note that DFT overestimates bond lengths; in fact, for PBE the calculated nearest-neighbor distances in the fcc lattice are *d* = 1.414 and 1.399 Å for Pt–Pt and Pd–Pd, respectively; however, for the purpose of calculating bond occurrences, this is not relevant. In general for both structures, Pt atoms are highly coordinated, within a range spanning 8 to 12 nearest neighbors for Dh and 9 to 12 for Th. Instead, Pd atoms are in general low-coordinate, but with a slight difference for the two structures. In Dh, Pd atoms can have 5 to 8 nearest neighbors, whereas for Th, the coordination number is either 6 or 7, with the only exception being the atom in the center of the NP, having 12 nearest neighbors. In particular, 24 Pd atoms have a coordination number equal to 6 in Th, whereas only 18 atoms have the same coordination in the Dh. A similar analysis was done for Pt_52_Pd_48_. The calculation of the number of bonds revealed that the Dh has 192 Pt–Pt bonds, 70 Pd–Pd bonds, and 177 mixed Pt–Pd bonds. The Th has instead 198 Pt–Pt bonds, 72 Pd–Pd bonds, and 168 Pt–Pd bonds. All 52 Pt atoms in Th have 9 to 12 nearest neighbors, whereas in Dh, 3 Pt atoms have 8 nearest-neighbors. In Dh, Pd atoms have 6 to 8 nearest neighbors, but in Th, only 6 or 7. In particular, the Dh has 21 Pd atoms with a coordination number equal to 6, whereas the Th has 24. Finally, we also analyzed Pt_104_Pd_76_. In this case, the Th has 432 Pt–Pt bonds, 120 Pd–Pd bonds, and 288 Pt–Pd bonds. The Dh has 418 Pt–Pt bonds, 115 Pd–Pd bonds, and 319 Pt–Pd bonds. The fcc structure has 409 Pt–Pt bonds, 107 Pd–Pd bonds, and 319 mixed Pt–Pd bonds. In Th, all 104 Pt atoms have 9 to 12 nearest neighbors, and Pd atoms have 6 to 9. In particular, a total of 24 Pd atoms have a coordination number equal to 6. In Dh, Pt atoms have 8 to 12 nearest neighbors, and Pd atoms have 6 to 8 nearest neighbors, with only 1 Pd atom in the core position having 12 nearest neighbors. A total of 23 Pd atoms have a coordination number equal to 6. Also in the fcc structure, Pt atoms have 9 to 12 nearest neighbors. Pd atoms have 6 to 8 nearest neighbors, and 3 of them in core positions have 12. A total of 26 Pd atoms have a coordination number equal to 6. All of these results are coherent with the surface segregation tendency of palladium atoms. However, it is difficult to establish a clear correlation between the different bond numbers or coordination numbers and the stability of tetrahedral structures. For example, in two out of the three cases considered here, the Th has a larger number of Pd atoms with the lowest possible coordination number of 6, the exception being Pt_104_Pd_76_. Similarly, in two of three cases, the tetrahedral structure has a larger number of Pt–Pt bonds. However, this is not the case for Pt_22_Pd_37_, suggesting that there are indeed other factors playing an important role in the final determination of the most stable structure. Therefore, we recommend looking for other stable tetrahedral Pt–Pd nanoparticles by following these two steps:

1. choosing the size according to eqs 10 and 11 that gives the number of atoms of irregular and regular truncated tetrahedra, respectively, and
2. choosing the optimal composition by filling the core with Pt atoms, the hexagonal islands and the triangular facets with Pd atoms, and eventually by putting excess Pt atoms inside the hexagonal islands.

## Conclusions

We showed that tetrahedral nanoparticles can be stabilized by alloying Pt and Pd. At 0 K, this was demonstrated both at the atomistic and ab initio levels, whereas the stability up to room temperature was proven only by atomistic calculations. The importance of our work is twofold. From a theoretical point of view, we proved the stability of the tetrahedral structural motif at the DFT level, up to a relatively large size (*N* = 180), showing that these structures can be recovered by a new series of magic numbers. From an experimental point of view, our results show that by mixing the two metals, one can in principle produce tetrahedral nanoparticles that are more stable than those made by pure metals, so they should be more resistant to aging under the action of a controlled environment and possibly less prone to shape changes during chemical reactions. In addition, we cannot exclude that the size limit of stable tetrahedral clusters can be further pushed forward since we have not yet proven the stability of other tetrahedral clusters that are next in the magic series. Moreover, we speculate that the effect of tetrahedral stabilization induced by the alloying of two metals can also be extended to other metal pairs, for which there are similar interactions between the two.

## Supporting Information Available

Basin hopping simulation parameters for global optimizations; energy differences between isomers for atomistic and DFT calculations; mixing energy calculations and plots; DFT normal modes calculations for Pt_2_Pd_4_; molecular dynamics simulations’ results; and link to an open-access repository containing the *XYZ* coordinates of all structures considered in this study (PDF)

## References

[ref1] Ferrando R. Structure and Properties of Nanoalloys; Frontiers of Nanoscience; Vol. 10; Elsevier, 2016.

[ref2] Baletto F.; Ferrando R.; Fortunelli A.; Montalenti F.; Mottet C. Crossover among structural motifs in transition and noble-metal clusters. J. Chem. Phys. 2002, 116, 3856 10.1063/1.1448484.

[ref3] Marks L. D. Experimental studies of small-particle structures. Rep. Prog. Phys. 1994, 57, 603–649. 10.1088/0034-4885/57/6/002.

[ref4] Cleveland C. L.; Landman U.; Schaaff T. G.; Shafigullin M. N.; Stephens P. W.; Whetten R. L. Structural Evolution of Smaller Gold Nanocrystals: The Truncated Decahedral Motif. Phys. Rev. Lett. 1997, 79, 1873–1876. 10.1103/PhysRevLett.79.1873.

[ref5] Teranishi T.; Kurita R.; Miyake M. Shape Control of Pt Nanoparticles. Journal of Inorganic and Organometallic Polymers 2000, 10, 145–156. 10.1023/A:1009476128466.

[ref6] Norimatsu F.; Mizokoshi Y.; Mori K.; Mizugaki T.; Ebitani K.; Kaneda K. Shape- and size-controlled synthesis of tetrahedral Pd nanoparticles using tetranuclear Pd cluster as precursor. Chem. Lett. 2006, 35, 276–277. 10.1246/cl.2006.276.

[ref7] Gracia-Pinilla M. A.; Pérez-Tijerina E.; Antúnez-García J.; Fernández-Navarro C.; Tlahuice-Flores A.; Mejía-Rosales S.; Montejano-Carrizales J. M.; José-Yacamán M. On the Structure and Properties of Silver Nanoparticles. J. Phys. Chem. C 2008, 112, 13492–13498. 10.1021/jp804085q.

[ref8] Yin A.-X.; Min X.-Q.; Zhang Y.-W.; Yan C.-H. Shape-Selective Synthesis and Facet-Dependent Enhanced Electrocatalytic Activity and Durability of Monodisperse Sub-10 nm Pt-Pd Tetrahedrons and Cubes. J. Am. Chem. Soc. 2011, 133, 3816–3819. 10.1021/ja200329p. 21348522

[ref9] Li Y.; Petroski J.; El-Sayed M. A. Activation Energy of the Reaction between Hexacyanoferrate(III) and Thiosulfate Ions Catalyzed by Platinum Nanoparticles. J. Phys. Chem. B 2000, 104, 10956–10959. 10.1021/jp002569s.

[ref10] Narayanan R.; El-Sayed M. A. Shape-Dependent Catalytic Activity of Platinum Nanoparticles in Colloidal Solution. Nano Lett. 2004, 4, 1343–1348. 10.1021/nl0495256.

[ref11] Narayanan R.; El-Sayed M. A. Catalysis with Transition Metal Nanoparticles in Colloidal Solution: Nanoparticle Shape Dependence and Stability. J. Phys. Chem. B 2005, 109, 12663–12676. 10.1021/jp051066p. 16852568

[ref12] Burda C.; Chen X.-B.; Narayanan R.; El-Sayed M. A. Chemistry and properties of nanocrystals of different shapes. Chem. Rev. 2005, 105, 1025–1102. 10.1021/cr030063a. 15826010

[ref13] Lu H. M.; Meng X. K. Theoretical Model to Calculate Catalytic Activation Energies of Platinum Nanoparticles of Different Sizes and Shapes. J. Phys. Chem. C 2010, 114, 1534–1538. 10.1021/jp9106475.

[ref14] Zheng G.; Carbó-Argibay E.; González-Romero E.; Pastoriza-Santos I.; Pérez-Juste J. Pd–Au Heteropentamers: Selective Growth of Au on Pd Tetrahedral Nanoparticles with Enhanced Electrocatalytic Activity. Cryst. Growth Des. 2020, 20, 5863–5867. 10.1021/acs.cgd.0c00500.

[ref15] Zheng P.; Paria D.; Wang H.; Li M.; Barman I. Optical properties of symmetry-breaking tetrahedral nanoparticles. Nanoscale 2020, 12, 832–842. 10.1039/C9NR08515G. 31830188 PMC7560971

[ref16] Tabatabaei M.; Sangar A.; Kazemi-Zanjani N.; Torchio P.; Merlen A.; Lagugné-Labarthet F. Optical Properties of Silver and Gold Tetrahedral Nanopyramid Arrays Prepared by Nanosphere Lithography. J. Phys. Chem. C 2013, 117, 14778–14786. 10.1021/jp405125c.

[ref17] Das P.; Chini T. K.; Pond J. Probing Higher Order Surface Plasmon Modes on Individual Truncated Tetrahedral Gold Nanoparticle Using Cathodoluminescence Imaging and Spectroscopy Combined with FDTD Simulations. J. Phys. Chem. C 2012, 116, 15610–15619. 10.1021/jp3047533.

[ref18] El koraychy E. y.; Roncaglia C.; Nelli D.; Cerbelaud M.; Ferrando R. Growth mechanisms from tetrahedral seeds to multiply twinned Au nanoparticles revealed by atomistic simulations. Nanoscale Horiz 2022, 7, 883–889. 10.1039/D1NH00599E. 35722927

[ref19] Li J.; Li X.; Zhai H.-J.; Wang L.-S. Au_20_: A Tetrahedral Cluster. Science 2003, 299, 864–867. 10.1126/science.1079879. 12574622

[ref20] Xia Y.; Nelli D.; Ferrando R.; Yuan J.; Li Z. Y. Shape control of size-selected naked platinum nanocrystals. Nat. Commun. 2021, 12, 3019 10.1038/s41467-021-23305-7. 34021147 PMC8139959

[ref21] Leary R. H.; Doye J. P. K. Tetrahedral global minimum for the 98-atom Lennard-Jones cluster. Phys. Rev. E 1999, 60, R6320–R6322. 10.1103/PhysRevE.60.R6320. 11970625

[ref22] Paz-Borbón L. O.; Mortimer-Jones T. V.; Johnston R. L.; Posada-Amarillas A.; Barcaro G.; Fortunelli A. Structures and energetics of 98 atom Pd-Pt nanoalloys: potential stability of the Leary tetrahedron for bimetallic nanoparticles. Phys. Chem. Chem. Phys. 2007, 9, 5202–5208. 10.1039/b707136a. 19459283

[ref23] Bruma A.; Ismail R.; Oliver Paz-Borbón L.; Arslan H.; Barcaro G.; Fortunelli A.; Li Z. Y.; Johnston R. L. DFT study of the structures and energetics of 98-atom AuPd clusters. Nanoscale 2013, 5, 646–652. 10.1039/C2NR32517A. 23223667

[ref24] Zhang H.; Jin M.; Xia Y. Enhancing the catalytic and electrocatalytic properties of Pt-based catalysts by forming bimetallic nanocrystals with Pd. Chem. Soc. Rev. 2012, 41, 8035–8049. 10.1039/c2cs35173k. 23080521

[ref25] Lim B.; Jiang M.; Camargo P. H. C.; Cho E. C.; Tao J.; Lu X.; Zhu Y.; Xia Y. Pd-Pt Bimetallic Nanodendrites with High Activity for Oxygen Reduction. Science 2009, 324, 1302–1305. 10.1126/science.1170377. 19443738

[ref26] Peng Z.; Yang H. Synthesis and Oxygen Reduction Electrocatalytic Property of Pt-on-Pd Bimetallic Heteronanostructures. J. Am. Chem. Soc. 2009, 131, 7542–7543. 10.1021/ja902256a. 19438286

[ref27] Wang L.; Nemoto Y.; Yamauchi Y. Direct Synthesis of Spatially-Controlled Pt-on-Pd Bimetallic Nanodendrites with Superior Electrocatalytic Activity. J. Am. Chem. Soc. 2011, 133, 9674–9677. 10.1021/ja202655j. 21619032

[ref28] Rossi G.; Ferrando R. Combining shape-changing with exchange moves in the optimization of nanoalloys. Computational and Theoretical Chemistry 2017, 1107, 66–73. 10.1016/j.comptc.2017.01.002.

[ref29] Rapetti D.; Roncaglia C.; Ferrando R. Optimizing the Shape and Chemical Ordering of Nanoalloys with Specialized Walkers. Advanced Theory and Simulations 2023, 6, 2300268 10.1002/adts.202300268.

[ref30] Rossi G.; Ferrando R.; Rapallo A.; Fortunelli A.; Curley B. C.; Lloyd L. D.; Johnston R. L. Global optimization of bimetallic cluster structures. I. Size-matched Ag-Pd, Ag-Au, and Pd-Pt systems. J. Chem. Phys. 2005, 122, 194309 10.1063/1.1898224. 16161575

[ref31] Giannozzi P.; et al. QUANTUM ESPRESSO: a modular and open-source software project for quantum simulations of materials. J. Phys.: Condens. Matter 2009, 21, 395502 10.1088/0953-8984/21/39/395502. 21832390

[ref32] Perdew J. P.; Burke K.; Ernzerhof M. Generalized gradient approximation made simple. Phys. Rev. Lett. 1996, 77, 3865–3868. 10.1103/PhysRevLett.77.3865. 10062328

[ref33] Parr R.; Yang W. Density-Functional Theory of Atoms and Molecules; Oxford University Press, 1994.

[ref34] Doye J. P. K.; Calvo F. Entropic Effects on the Size Dependence of Cluster Structure. Phys. Rev. Lett. 2001, 86, 3570–3573. 10.1103/PhysRevLett.86.3570. 11328025

[ref35] Doye J. P. K.; Wales D. J.; Berry R. S. The effect of the range of the potential on the structures of clusters. J. Chem. Phys. 1995, 103, 4234 10.1063/1.470729.

[ref36] Manninen K.; Manninen M. Stacking faults in close-packed clusters. European Physical Journal D - Atomic, Molecular, Optical and Plasma Physics 2002, 20, 243–249. 10.1140/epjd/e2002-00117-0.

[ref37] De Clercq A; Giorgio S; Mottet C Pd surface and Pt subsurface segregation in Pt_1–*c*_Pd_*c*_ nanoalloys. J. Phys.: Condens. Matter 2016, 28, 064006 10.1088/0953-8984/28/6/064006. 26795206

[ref38] Nelli D.; Krishnadas A.; Ferrando R.; Minnai C. One-Step Growth of Core–Shell (PtPd)@Pt and (PtPd)@Pd Nanoparticles in the Gas Phase. J. Phys. Chem. C 2020, 124, 14338–14349. 10.1021/acs.jpcc.0c02621.

[ref39] Nelli D.; Roncaglia C.; Ahearn S.; Di Vece M.; Ferrando R.; Minnai C. Octahedral Growth of PtPd Nanocrystals. Catalysts 2021, 11, 718 10.3390/catal11060718.

[ref40] Samsonov V.; Romanov A.; Talyzin I.; Lutsay A.; Zhigunov D.; Puytov V. Puzzles of Surface Segregation in Binary Pt-Pd Nanoparticles: Molecular Dynamics and Thermodynamic Simulations. Metals 2023, 13, 1269 10.3390/met13071269.

[ref41] Rousset J. L.; Cadrot A. M.; Cadete Santos Aires F. J.; Renouprez A.; Mélinon P.; Perez A.; Pelarin M.; Vialle J. L.; Broyer M. Study of bimetallic Pd-Pt clusters in both free and supported phases. J. Chem. Phys. 1995, 102, 8574–8585. 10.1063/1.468847.

[ref42] Rousset J. L.; Renouprez A. J.; Cadrot A. M. Ion-scattering study and Monte Carlo simulations of surface segregation in Pd-Pt nanoclusters obtained by laser vaporization of bulk alloys. Phys. Rev. B 1998, 58, 2150–2156. 10.1103/PhysRevB.58.2150.

[ref43] Fiermans L.; De Gryse R.; De Doncker G.; Jacobs P. A.; Martens J. A. Pd Segregation to the Surface of Bimetallic Pt-Pd Particles Supported on H-β Zeolite Evidenced with X-Ray Photoelectron Spectroscopy and Argon Cation Bombardment. J. Catal. 2000, 193, 108–114. 10.1006/jcat.2000.2868.

[ref44] Bernardi F.; Alves M. C. M.; Traverse A.; Silva D. O.; Scheeren C. W.; Dupont J.; Morais J. Monitoring Atomic Rearrangement in Pt_*x*_Pd_1–*x*_ (x = 1, 0.7, or 0.5) Nanoparticles Driven by Reduction and Sulfidation Processes. J. Phys. Chem. C 2009, 113, 3909–3916. 10.1021/jp805465x.

