# Supplementary material for: Tetrahedral Clusters Stabilized by Alloying
Source: J Phys Chem A. 2023 Dec 19;128(1):89–96. doi: 10.1021/acs.jpca.3c06033 (PMC10788904; doi:10.1021/acs.jpca.3c06033)
Supplement: Supplementary file 1 — jp3c06033_si_001.pdf [file jp3c06033_si_001.pdf]

Supporting Information: Tetrahedral Clusters Stabilized by Alloying

Cesare Roncaglia† and Riccardo Ferrando*,‡,¶

†*Dipartimento di Fisica dell'Università di Genova, via Dodecaneso 33, Genova 16146, Italy*

‡*Dipartimento di Fisica dell'Università di Genova and CNR-IMEM, via Dodecaneso 33, Genova 16146, Italy*

¶*Corresponding author*

E-mail: ferrando@fisica.unige.it

Basin Hopping simulation parameters

Here we report simulation parameters of global optimization searches. Most simulations were performed by the basin hopping algorithm using the same approach of Refs.[1,2] In the difficult case of $Pt_{104}Pd_{76}$ we used the recently developed Flying-Landing-Hiking (FLH) algorithm[3] which is more efficient than standard basin hopping. In FLH, three basin-hopping walkers are used, each with a specific task in the exploration of the landscape. Different types of moves were used: shake, Brownian and random exchange moves. These moves are described in Ref.[1] In the following tables, temperatures are in K.

N=59

Table S1: BH simulation parameters for Pt_0Pd_{59}.

Pt_0Pd_{59}				
	S1	S2	S3	S4
seeded	no	no	yes	yes
MC steps	$5 \cdot 10^5$	$5 \cdot 10^5$	$5 \cdot 10^5$	$5 \cdot 10^5$
% Bonds	20	0	0	0
Bonds acc Temp	300			
% Shake	60	80	0	0
Shake acc Temp	1500	1500		
% Brownian	20	20	100	100
Brownian acc Temp	1500	1500	1500	1500
Brownian steps	500	500	500	500
Brownian Temp	2000	2000	2000	2000

Table S2: BH simulation parameters for $Pt_{22}Pd_{37}$ and $Pt_{23}Pd_{36}$.

$Pt_{22}Pd_{37}$, $Pt_{23}Pd_{36}$								
	S1	S2	S3	S4	S5	S6	S7	S8
seeded	no	yes	yes	yes	yes	yes	yes	yes
MC steps	$5 \cdot 10^5$	$5 \cdot 10^5$	$5 \cdot 10^5$	$5 \cdot 10^5$	$5 \cdot 10^5$	$5 \cdot 10^5$	$5 \cdot 10^5$	$5 \cdot 10^5$
% Bonds	0	0	0	0	0	0	0	0
Bonds acc Temp								
% Shake	60	0	0	0	0	0	0	0
Shake acc Temp	1500							
% Brownian	10	0	20	20	20	0	0	0
Brownian acc Temp	1500		1500	1500	1500			
Brownian steps	500		500	500	500			
Brownian Temp	2000		2000	2000	2000			
% Exchange	30	100	80	80	80	100	100	100
Exchange acc Temp	100	100	100	100	100	100	100	100

Table S3: BH simulation parameters for $Pt_{24}Pd_{35}$.

$Pt_{24}Pd_{35}$		
	S1	S2
seeded	no	yes
MC steps	$5 \cdot 10^5$	$5 \cdot 10^5$
% Bonds	0	0
Bonds acc Temp		
% Shake	60	0
Shake acc Temp	1500	
% Brownian	10	0
Brownian acc Temp	1500	
Brownian steps	500	
Brownian Temp	2000	
% Exchange	30	100
Exchange acc Temp	100	100

Table S4: BH simulation parameters for $Pt_{35}Pd_{24}$.

$Pt_{35}Pd_{24}$				
	S1	S2	S3	S4
seeded	no	yes	yes	yes
MC steps	$5 \cdot 10^5$	$5 \cdot 10^5$	$5 \cdot 10^5$	$5 \cdot 10^5$
% Bonds	0	0	0	0
Bonds acc Temp				
% Shake	60	0	0	60
Shake acc Temp	1500			1500
% Brownian	10	0	0	10
Brownian acc Temp	1500			1500
Brownian steps	500			500
Brownian Temp	2000			2000
% Exchange	30	100	100	30
Exchange acc Temp	100	100	100	100

Table S5: BH simulation parameters for $Pt_{59}Pd_0$.

$Pt_{59}Pd_0$		
	S1	S2
seeded	no	yes
MC steps	$5 \cdot 10^5$	$5 \cdot 10^5$
% Bonds	20	0
Bonds acc Temp	300	
% Shake	60	0
Shake acc Temp	1500	
% Brownian	20	100
Brownian acc Temp	1500	1500
Brownian steps	500	500
Brownian Temp	2000	2000

N=100

Table S6: BH simulation parameters for Pt_0Pd_{100}.

Pt_0Pd_{100}		
	S1	S2
seeded	no	yes
MC steps	$1 \cdot 10^6$	$5 \cdot 10^5$
% Bonds	30	20
Bonds acc Temp	300	300
% Shake	50	0
Shake acc Temp	1500	
% Brownian	20	80
Brownian acc Temp	2000	1500
Brownian steps	250	500
Brownian Temp	2000	2000

Table S7: BH simulation parameters for $Pt_{36}Pd_{64}$ and $Pt_{40}Pd_{60}$.

$Pt_{36}Pd_{64}$, $Pt_{40}Pd_{60}$				
	S1	S2	S3	S4
seeded	no	yes	yes	yes
MC steps	$1 \cdot 10^6$	$5 \cdot 10^5$	$5 \cdot 10^5$	$5 \cdot 10^5$
% Bonds	0	0	0	20
Bonds acc Temp				300
% Shake	40	0	0	0
Shake acc Temp	1500			
% Brownian	30	20	20	20
Brownian acc Temp	1500	1500	1500	2000
Brownian steps	500	500	500	300
Brownian Temp	2000	2000	2000	2000
% Exchange	30	80	80	60
Exchange acc Temp	100	100	100	100

Table S8: BH simulation parameters for $Pt_{48}Pd_{52}$.

$Pt_{48}Pd_{52}$					
	S1	S2	S3	S4	S5
seeded	no	no	yes	yes	yes
MC steps	$1 \cdot 10^6$	$5 \cdot 10^5$	$5 \cdot 10^5$	$5 \cdot 10^5$	$5 \cdot 10^5$
% Bonds	0	20	0	0	10
Bonds acc Temp		300			300
% Shake	40	20	0	0	0
Shake acc Temp	1500	1500			
% Brownian	30	30	20	20	10
Brownian acc Temp	1500	1500	1500	1500	2000
Brownian steps	500	250	500	500	300
Brownian Temp	2000	2000	2000	2000	2000
% Exchange	30	30	80	80	80
Exchange acc Temp	100	100	100	100	100

Table S9: BH simulation parameters for $Pt_{52}Pd_{48}$.

$Pt_{52}Pd_{48}$				
	S1	S2	S3	S4
seeded	no	yes	yes	yes
MC steps	$1 \cdot 10^6$	$5 \cdot 10^5$	$5 \cdot 10^5$	$5 \cdot 10^5$
% Bonds	0	0	0	10
Bonds acc Temp				300
% Shake	40	0	0	0
Shake acc Temp	1500			
% Brownian	30	20	20	10
Brownian acc Temp	1500	1500	1500	2000
Brownian steps	500	500	500	300
Brownian Temp	2000	2000	2000	2000
% Exchange	30	80	80	80
Exchange acc Temp	100	100	100	100

Table S10: BH simulation parameters for $Pt_{100}Pd_0$. Eight independent simulations were performed. The lowest-energy tetrahedral structures was found in 2 simulations. In the remaining simulations, fcc, decahedral and twin structures were found. These structures are very close in energy to the tetrahedral one (differences in the range 0.01-0.04 eV).

$Pt_{100}Pd_0$	
	S1-8
seeded	no
MC steps	$1 \cdot 10^5$
% Bonds	20
Bonds acc Temp	500
% Brownian	80
Brownian acc Temp	3000
Brownian steps	200
Brownian Temp	3000

N=180

Table S11: BH simulation parameters for Pt_0Pd_{180}.

Pt_0Pd_{180}				
	S1	S2	S3	S4
seeded	no	yes	yes	yes
MC steps	$1 \cdot 10^6$	$5 \cdot 10^5$	$5 \cdot 10^5$	$5 \cdot 10^5$
% Bonds	20	20	20	0
Bonds acc Temp	300	300	300	
% Shake	20	10	10	20
Shake acc Temp	1500	1500	1500	
% Brownian	60	70	70	80
Brownian acc Temp	1500	1500	1500	2000
Brownian steps	500	500	500	250
Brownian Temp	2000	2000	2000	2000

Table S12: BH simulation parameters for $Pt_{80}Pd_{100}$.

$Pt_{80}Pd_{100}$										
	S1	S2	S3	S4	S5	S6	S7	S8	S9	S10
seeded	no	no	yes	yes	yes	yes	yes	yes	yes	yes
MC steps	$1 \cdot 10^6$	$1 \cdot 10^6$	$5 \cdot 10^5$	$5 \cdot 10^5$	$5 \cdot 10^5$	$5 \cdot 10^5$	$5 \cdot 10^5$	$5 \cdot 10^5$	$5 \cdot 10^5$	$5 \cdot 10^5$
% Bonds	0	0	0	0	0	0	0	10	0	0
Bonds acc Temp								300		
% Shake	40	40	0	0	0	0	20	0	0	0
Shake acc Temp	1500	1500					1500			
% Brownian	30	30	0	20	20	0	40	60	0	0
Brownian acc Temp	1500	1500		1500	1500		1500	2000		
Brownian steps	500	500		500	500		500	5000		
Brownian Temp	2000	2000		2000	2000		2000	2000		
% Exchange	30	30	100	80	80	100	40	30	100	100
Exchange acc Temp	100	100	100	100	100	100	100	100	100	100

Table S13: BH simulation parameters for $Pt_{104}Pd_{76}$. The FLH algorithm was used.[3] The columns F, L, and H refer to the Flying, Landing, and Hiking walkers, respectively. Four independent simulations of 2 10^6 steps were made. In the simulations, each walker performed one third of the steps. The global minimum (apart from the swap of a one pair of atoms) was successfully found only in one of these simulations.

$Pt_{104}Pd_{76}$			
	F	L	H
seeded	no	no	no
% Brownian	80	20	20
Brownian acc Temp	3500	300	300
Brownian steps	200	200	200
Brownian Temp	3500	2000	2000
% Exchange	20	80	80
Exchange acc Temp	500	100	100

Table S14: BH simulation parameters for $Pt_{180}Pd_0$.

$Pt_{180}Pd_0$			
	S1	S2	S3
seeded	no	yes	yes
MC steps	$1 \cdot 10^6$	$5 \cdot 10^5$	$5 \cdot 10^5$
% Bonds	20	20	0
Bonds acc Temp	300	300	
% Shake	20	10	20
Shake acc Temp	1500	1500	1500
% Brownian	60	70	80
Brownian acc Temp	1500	1500	1500
Brownian steps	500	500	500
Brownian Temp	2000	2000	2000

Energy differences for plot in Figure 5

Table S15: Energy comparisons (in eV) between Pt-Pd decahedral (Dh), tetrahedral (Th) and face-centered cubic (fcc) isomers for different sizes and compositions, both for Gupta and DFT. The best structures are referenced by 0.

**N=59**									
	Pt_0Pd_{59}			$Pt_{22}Pd_{37}$			$Pt_{23}Pd_{36}$		
	Gupta	PBE	LDA	Gupta	PBE	LDA	Gupta	PBE	LDA
Dh	0.0455	0	0	0.939	0.0826	0.222	0.881	0.137	0.238
Th	0	0.224	0.0541	0	0	0	0	0	0
	$Pt_{24}Pd_{35}$			$Pt_{35}Pd_{24}$			$Pt_{59}Pd_0$		
Dh	0.748	0	0.0757	0.156	0	0	0	0	0
Th	0	0.0193	0	0	0.215	0.162	0.0426	1.89	1.84
**N=100**									
	Pt_0Pd_{100}			$Pt_{36}Pd_{64}$			$Pt_{40}Pd_{60}$		
	Gupta	PBE	LDA	Gupta	PBE	LDA	Gupta	PBE	LDA
Dh	0	0.0288	0.0636	0	0.520	0.407	0	0.553	0.462
Th	0.0346	0	0	0.0201	0	0	0.140	0	0
	$Pt_{48}Pd_{52}$			$Pt_{52}Pd_{48}$			$Pt_{100}Pd_0$		
Dh	0	0.488	0.518	0	0.383	0.339	0.0418	0	0
Th	0.0841	0	0	0.0457	0	0	0	0.261	0.185

**N=180**						
	Pt_0Pd_{180}			$Pt_{80}Pd_{100}$		
	Gupta	PBE	LDA	Gupta	PBE	LDA
Dh	0	0.674	0.731	0.105	1.15	1.17
Th	0.128	0.856	1.02	0	0.451	0.556
Fcc/twin	0.214	0	0	0.724	0	0
	$Pt_{104}Pd_{76}$			$Pt_{180}Pd_0$		
Dh	0.146	0.576	0.418	0	1.61	1.75
Th	0	0	0	0.170	3.47	3.75
Fcc/twin	0.528	0.426	0.473	0.138	0	0

Mixing energy

The energetic stability of nanoalloys can be analyzed by means of the mixing energy E_{mix}, defined as

$$E_{\text{mix}}(m,n) = E(m,n) - \frac{m}{N}E(N,0) - \frac{n}{N}E(0,N) \quad (1)$$

where $E(m,n)$ is the binding energy of a nanoalloys having m atoms of one type and n atoms of the other. In our case m will be the number of Pt atoms in the Pt-Pd nanoalloys. We calculated mixing energy differences between tetrahedral and decahedral structures for all compositions studied at $N = 59$ and $N = 100$, and mixing energy differences between tetrahedral and fcc motifs for the case $N = 180$. The results are plotted in Figures S1, S2 and S3.

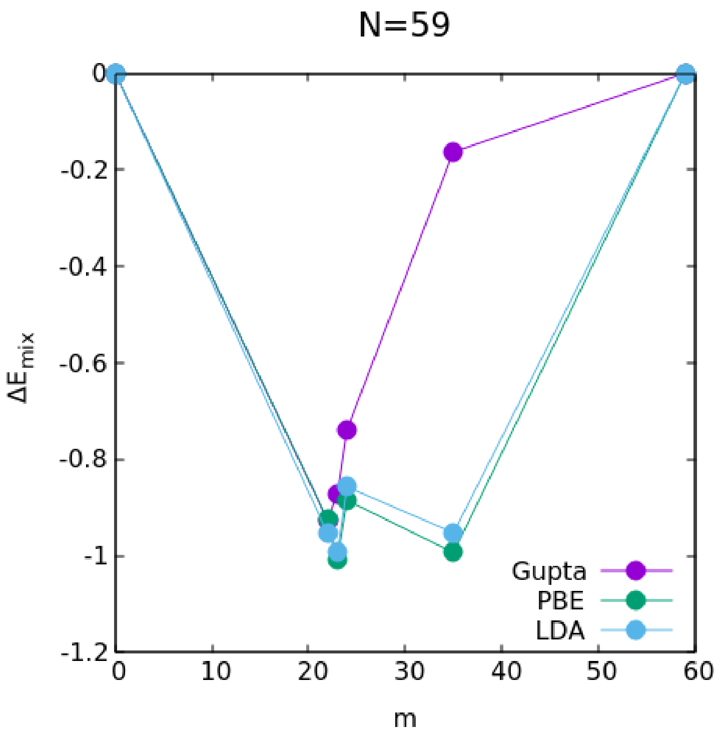

Figure S1: Mixing energy differences $\Delta E_{\text{mix}} = E^{\text{Th}}_{\text{mix}} - E^{\text{Dh}}_{\text{mix}}$ for $N = 59$ Pt-Pd nanoalloys.

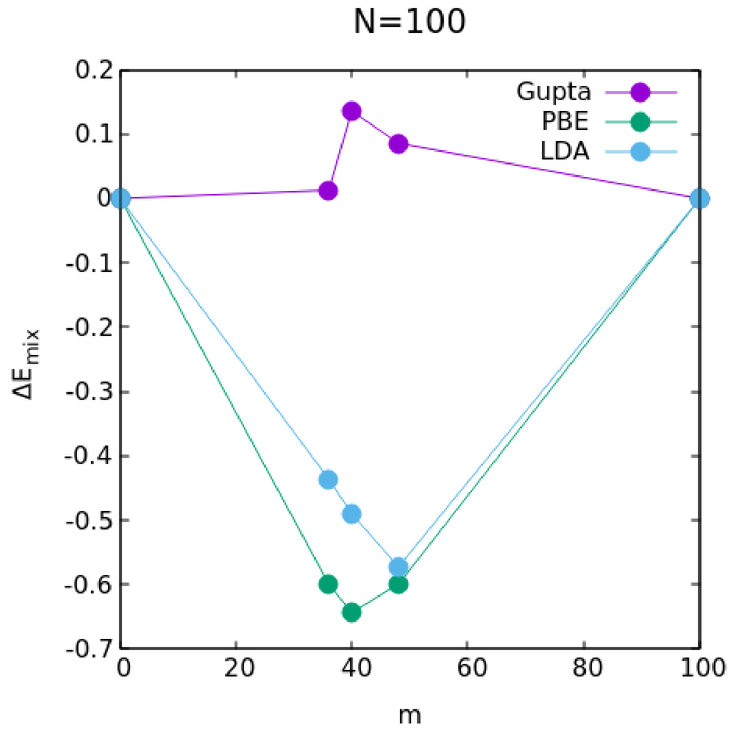

Figure S2: Mixing energy differences $\Delta E_{\rm mix} = E_{\rm mix}^{\rm Th} - E_{\rm mix}^{\rm Dh}$ for $N = 100$ Pt-Pd nanoalloys.

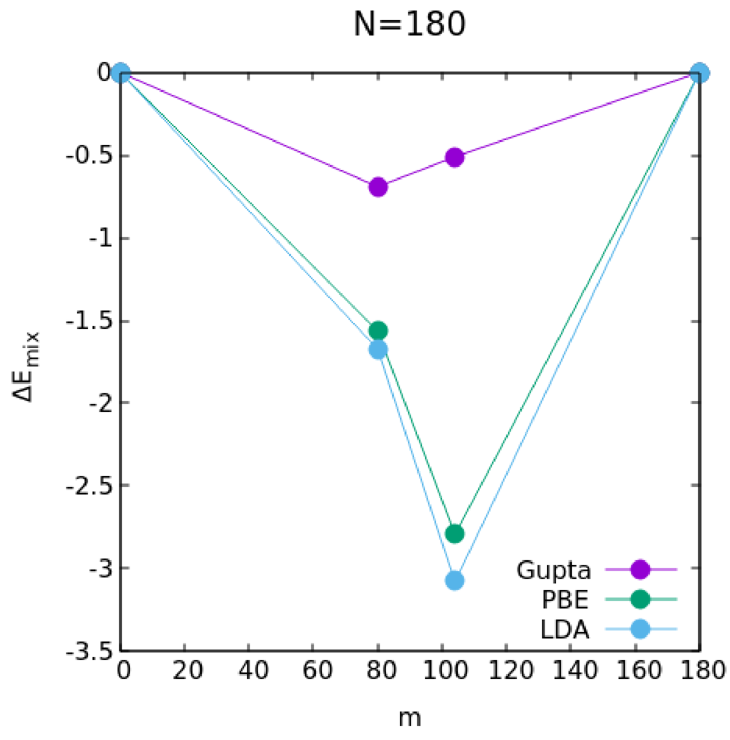

Figure S3: Mixing energy differences $\Delta E_{\rm mix} = E_{\rm mix}^{\rm Th} - E_{\rm mix}^{\rm fcc}$ for $N = 180$ Pt-Pd nanoalloys.

Normal modes for Pt_2Pd_4

We calculated normal modes for a small Pt_2Pd_4 cluster to compare both atomistic and ab-initio results. The optimal structure for Pt_2Pd_4 cluster was first found by global optimization with the Gupta potential. This structure is shown in Fig. S4. From this structure normal

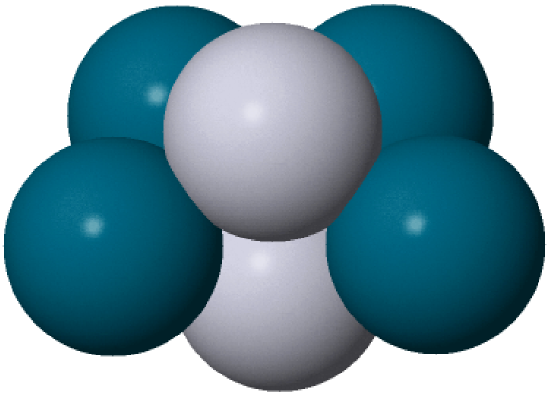

Figure S4: Structure of Pt_2Pd_4.

modes are calculated with the Gupta potential by diagonalization of the dynamical matrix. For ab-initio calculations, the structure was first relaxed with **pw.x** program of Quantum Espresso, using low convergence thresholds (10^{-6} Ry for total energy and 10^{-6} Ry/a.u. for total force, 10^{-15} Ry for electron self-consistent calculations). K-points were sampled using a 5x5x5 Monkhorst-Pack grid. Finally, the program **ph.x** was used for estimating frequencies of normal modes at gamma point. In the Table S16 we report the 12 non-zero normal modes, units are in 10^{12} rad/s. There is in general a good agreement between normal modes values.

Table S16: Normal modes vibrations as calculated by Gupta and DFT.

	Gupta	DFT
ω_1	7.15	12.3
ω_2	10.7	15.1
ω_3	14.4	16.1
ω_4	16.5	17.7
ω_5	18.0	19.1
ω_6	21.8	19.9
ω_7	23.7	21.3
ω_8	23.8	24.0
ω_9	24.4	28.4
ω_{10}	32.4	32.1
ω_{11}	39.4	36.6
ω_{12}	47.2	41.4

Molecular Dynamics simulations

In order to better demonstrate the stability of tetrahedral structures at temperatures higher than 0K, we also performed three molecular dynamics simulations at a constant temperature of 400K for all the three structures considered in free energy calculations in the main text. In particular, we considered $Pt_{22}Pd_{37}$ for N=59, $Pt_{36}Pd_{64}$ for N=100 and $Pt_{104}Pd_{76}$ for N=180. For all simulations, the number of time steps used for propagating the trajectories was fixed to $2{\cdot}10^8$. The value of the time step was set to 5 fs, so that all simulations lasted $1\mu s$. We used interatomic forces derived from the Gupta potential as described in the main text, the velocity-Verlet algorithm for integrating the equations of motion and the Andersen thermostat to simulate the canonical ensemble properties. For all three simulations, we used the tetrahedral structure as initial configuration. In all cases, no transitions to other structures were observed. This demonstrate that, within the approximation given by the atomistic potential, tetrahedral structure are stable at 400K for at least $1\mu s$. We show in Figures S5, S6 and S7 the evolution of the total energy as a function of time for the three molecular dynamics simulations.

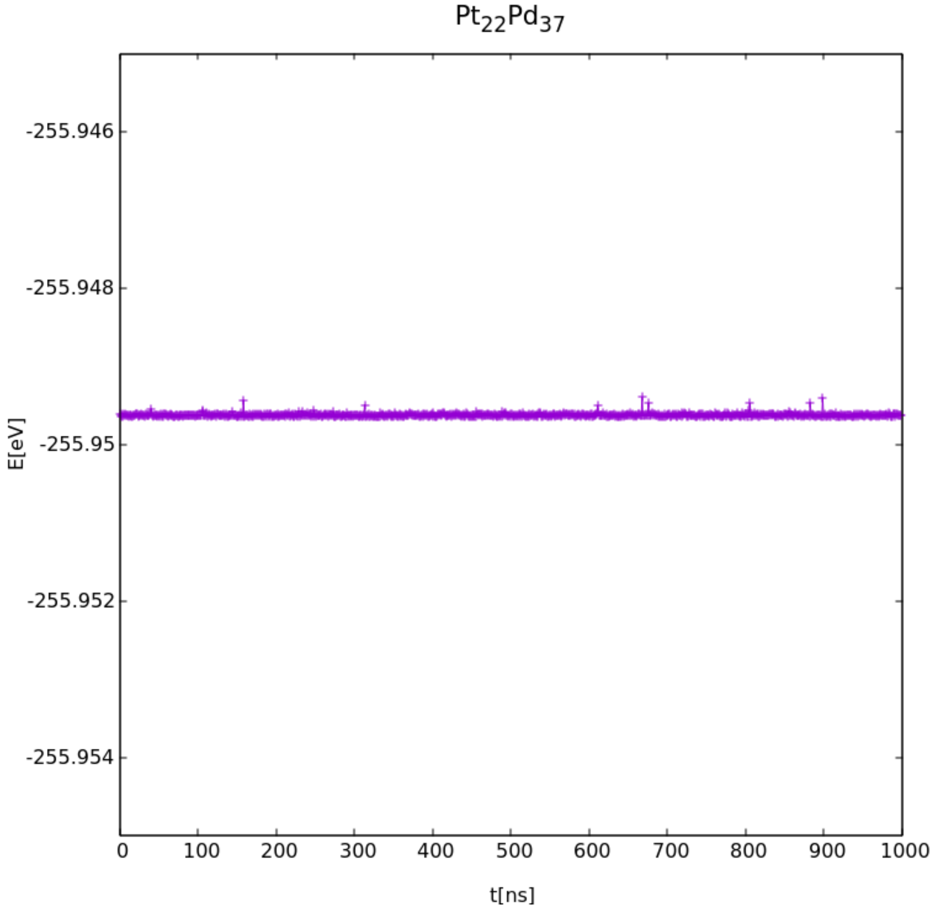

Figure S5: Total energy of $Pt_{22}Pd_{37}$ tetrahedron as a function of time.

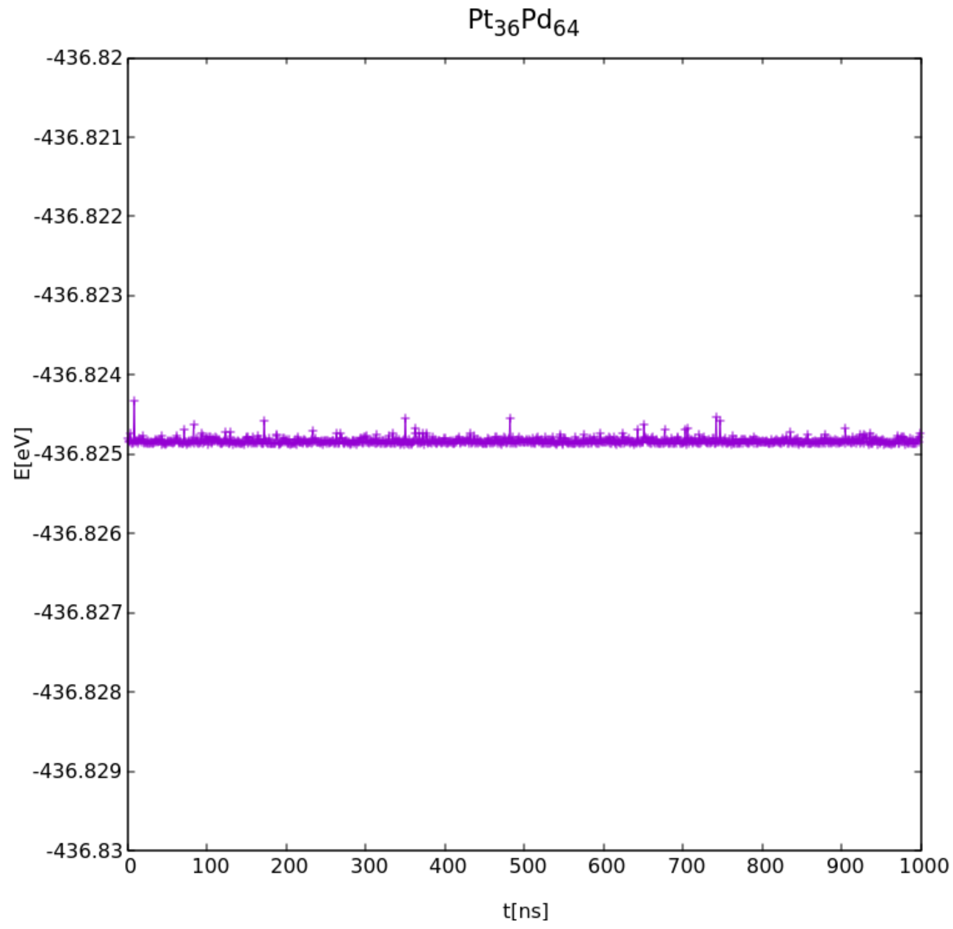

Figure S6: Total energy of $Pt_{36}Pd_{64}$ tetrahedron as a function of time.

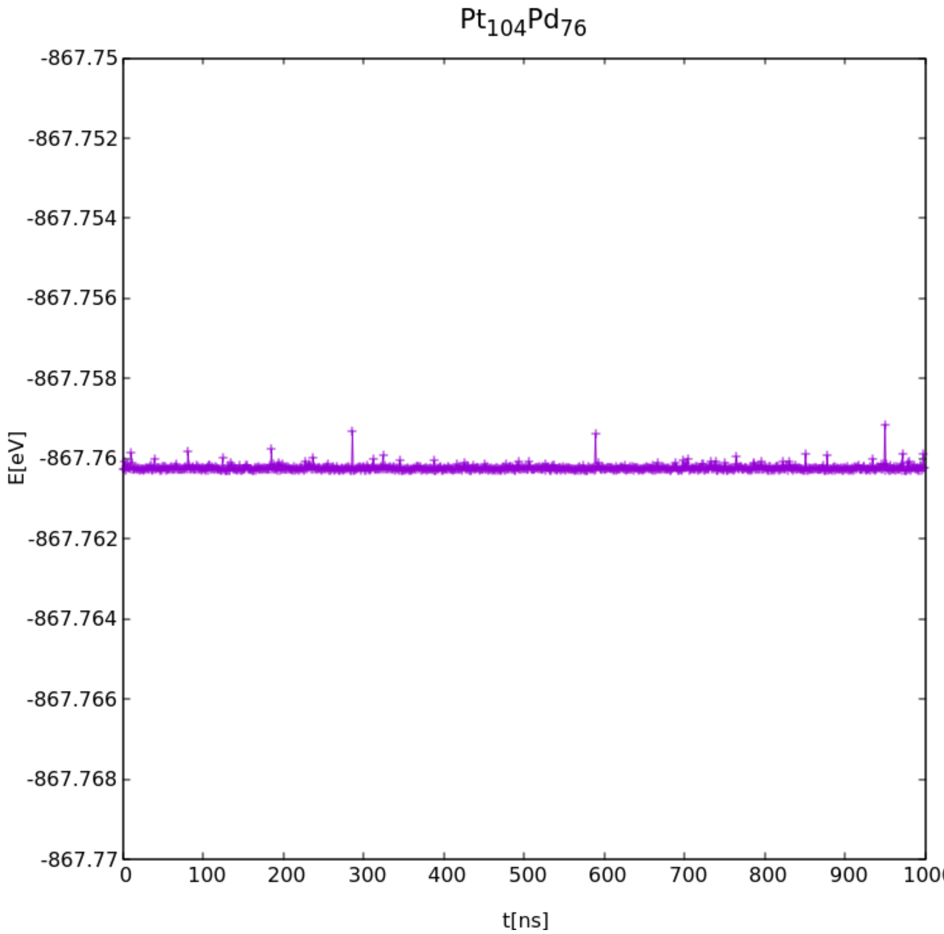

Figure S7: Total energy of $Pt_{104}Pd_{76}$ tetrahedron as a function of time.

XYZ coordinates of global minima

XYZ coordinates of all structures considered for Gupta and DFT calculations are available at the following open-access Zenodo repository: `https://zenodo.org/records/10066347`.

References

(1) Rossi, G.; Ferrando, R. Searching for low-energy structures of nanoparticles: a comparison of different methods and algorithms. *J. Phys. Cond. Mat.* **2009**, *21*, 084208.

(2) Rossi, G.; Ferrando, R. Combining shape-changing with exchange moves in the optimization of nanoalloys. *Computational and Theoretical Chemistry* **2017**, *1107*, 66 – 73.

(3) Rapetti, D.; Roncaglia, C.; Ferrando, R. Optimizing the Shape and Chemical Ordering of Nanoalloys with Specialized Walkers. *Advanced Theory and Simulations* **2023**, *6*, 2300268.
